# The Roles of Cullins E3 Ubiquitin Ligases in the Lipid Biosynthesis of the Green Microalgae *Chlamydomonas reinhardtii*

**DOI:** 10.3390/ijms22094695

**Published:** 2021-04-29

**Authors:** Qiulan Luo, Xianghui Zou, Chaogang Wang, Yajun Li, Zhangli Hu

**Affiliations:** 1 School of Life Sciences and Food Engineering, Hanshan Normal University, Chaozhou 521041, China; luoqiulan_79@163.com (Q.L.); xzhui@hstc.edu.cn (X.Z.); 2 Guangdong Technology Research Center for Marine Algal Bioengineering, Guangdong Provincial Key Laboratory for Plant Epigenetics, Shenzhen Engineering Laboratory for Marine Algal Biotechnology, Longhua Innovation Institute for Biotechnology, College of Life Sciences and Oceanography, Shenzhen University, Shenzhen 518060, China; charlesw@szu.edu.cn; 3 Hainan Provincial Key Laboratory for Functional Components Research and Utilization of Marine Bio-Resources, Institute of Tropical Bioscience and Biotechnology, Hainan Academy of Tropical Agricultural Resource, Chinese Academy of Tropical Agricultural Sciences, Haikou 517010, China; liyajun_1980@163.com

**Keywords:** *Chlamydomonas reinhardtii*, cullin, lipid metabolic, RNA inference, fatty acid

## Abstract

Microalgae-based biodiesel production has many advantages over crude oil extraction and refinement, thus attracting more and more concern. Protein ubiquitination is a crucial mechanism in eukaryotes to regulate physiological responses and cell development, which is highly related to algal biodiesel production. Cullins as the molecular base of cullin-RING E3 ubiquitin ligases (CRLs), which are the largest known class of ubiquitin ligases, control the life activities of eukaryotic cells. Here, three cullins (CrCULs) in the green microalgae *Chlamydomonas reinhardtii* were identified and characterized. To investigate the roles of CrCULs in lipid metabolism, the gene expression profiles of *CrCUL*s under nutrition starvation were examined. Except for down-regulation under nitrogen starvation, the *CrCUL3* gene was induced by sulfur and iron starvation. *CrCUL2* seemed insensitive to nitrogen and sulfur starvation because it only had changes after treatment for eight days. *CrCUL4* exhibited an expression peak after nitrogen starvation for two days but this declined with time. All *CrCUL*s expressions significantly increased under iron deficiency at two and four days but decreased thereafter. The silencing of *CrCUL2* and *CrCUL4* expression using RNAi (RNA interference) resulted in biomass decline and lipids increase but an increase of 20% and 28% in lipid content after growth for 10 days, respectively. In *CrCUL2* and *CrCUL4* RNAi lines, the content of fatty acids, especially C16:0 and C18:0, notably increased as well. However, the lipid content and fatty acids of the *CrCUL3* RNAi strain slightly changed. Moreover, the subcellular localization of CrCUL4 showed a nuclear distribution pattern. These results suggest CrCUL2 and CrCUL4 are regulators for lipid accumulation in *C. reinhardtii*. This study may offer an important complement of lipid biosynthesis in microalgae.

## 1. Introduction

Biodiesel from microalgae as a third generation biofuel has attracted growing attention because of its unique advantages such as a high efficiency for photosynthesis, the utilization of wastewater and the occupancy of less land [1]. Culture cost constitutes a large part of microalgae biodiesel production; therefore, numerous research has been performed to enhance the lipid and biomass production in microalgae by genetic engineering. Under abiotic stresses such as the deprivation of nutrients, microalgae accumulate lipids in the form of triacylglycerol (TAG), which is the main component of biodiesel [2]. To date, two TAG synthesis pathways have been found in microalgae; i.e., the Kennedy pathway and phospholipid: diacylglycerol acyltransferase (PDAT) mediated TAG synthesis [3]. In *C. reinhardtii*, the key enzymes involved in the Kennedy pathway were revealed in prior years including diacylglycerol acyltransferase (DGAT), glycerol-3-phosphate acyltransferase (GPAT) and Glycerol-3-phosphate dehydrogenases (GPDH) [4,5,6]. Increasing or silencing the expression of the above genes has been applied to improve lipid content and indeed made certain achievements. For example, DGAT homologue genes, which have been demonstrated to play important roles in the TAG accumulation in higher plants, have distinct roles in microalgae. The overexpression of five *Chlamydomonas DGAT2* genes showed only CrDGAT2-1 and CrDGAT2-5 could slightly increase the TAG content in cells [7]. An enhanced mRNA expression of three type 2 *DGAT* genes changed neither the lipid content nor fatty acid profiles [8]. The knockdown or overexpression of five *GPDH* homolog genes showed that only *GPDH2* and *GPDH3* transgenetic lines had significant lipid concentration and composition changes [4]. In addition, the enzymes regulating fatty acid metabolism such as acyl-ACP thioesterase (Fat), acetyl-CoA carboxylase (ACCase) and phosphoenolpyruvate carboxylase (PEPC) were also identified and proven to participate in biosynthesis of TAGs. The overexpression or silencing of these lipid metabolism-related genes in microalgae could change lipid contents and compositions often accompanied by a lower biomass and chlorophyll concentration, which indicated brakes in the biodiesel yield [9,10,11]. Therefore, in order to promote microalgae biodiesel production, it is necessary to uncover the mechanism of microalgae lipid biosynthesis.

Genomics, transcriptomics, proteomics and metabolomics analyses of *C. reinhardtii*, which is a model species for microalgae research, suggest that the TAG accumulation in microalgae is a complicated network system including key enzymes of lipid biosynthesis and transcriptional and post-transcriptional regulators [12,13]. The ubiquitin proteasome pathway (UPP) is a major regulatory network that permits eukaryote cells to integrate signals into a cellular response. In the UPP, highly conserved 76-amino acid protein ubiquitin (Ub) forms polymers by a three step (E1, E2 and E3) conjugation [14]. E3 ubiquitin ligases (E3s) confer the substrate specificity in ubiquitination. E3s have diverse functions in the eukaryote cell including the adjustment of the biosynthesis of lipids. It has been reported that in adipose tissue, E3 ubiquitin ligase constitutive photomorphogenic protein 1 (COP1) directly degrades ACCase, the rate-limiting enzyme in fatty acid synthesis, triggered by pseudokinase Tribbles 3 (TRB3) [15]. The survey of lipid droplets (LD) also showed that E3 ubiquitin ligase acts in an important regulatory way to control LD formation [16]. However, the current understanding of the roles of E3s in microalgae lipid metabolism is very limited.

**Figure 1:**
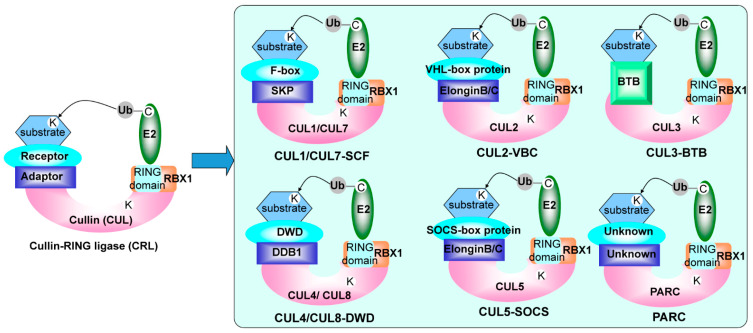
Diagram of cullin-RING ligases [17]. The C in the E2 presents the active site cysteine that binds to activated ubiquitin (Ub). The K in substrates and cullins indicates the acceptor sites for ubiquitin or Nedd8, respectively.

Cullin assembles cullin-RING E3 ubiquitin ligase (CRL) complexes, which are the largest family of E3 ligases with more than 200 members [17]. The cullin family evolutionarily conserves in different species; e.g., there are seven human cullins, five Arabidopsis cullins and three yeast cullins [18]. In CRL complexes, cullin proteins often tether both a substrate-recognition subunit (SRS) through an adaptor protein and the RING finger component, which recruits an E2 ubiquitin-conjugating enzyme (Figure 1). CUL1 and CUL7 interact with SKP1 (S-phase kinase-associated protein 1) and F-box, commonly known as SCF, recruiting substrates through the adaptor protein SKP1 and F-box protein [19,20]. CUL2 and CUL5 bind to the elongin B/C in the suppressor of cytokine signaling/elongin-B/C (SOCS/B/C) boxes. CUL3 is specifically merged into a single ‘Broad complex, Tramtrack, Bric-a-brac’ (BTB) domain containing a polypeptide that assembles the adaptor and substrate receptor [21]. CUL4 links the substrate through the large protein DNA damage-binding protein-1 (DDB1), which binds to SRSs containing WD repeats of a subclass called ‘DWD’ [22]. Due to the reaction with RING Box 1 (RBX1) and modified by Nedd8, the PARkin-like cytoplasmic protein (PARC) was defined as a cullin protein but its function is still unknown [16]. More mechanisms and roles of cullins in eukaryote cells need to be revealed in future.

In *C. reinhardtii*, ubiquitin has been reported to play similar roles in response to various stresses. Using a yeast two-hybrid screening assay, CrCUL4 has been proven to interact with CrDDB1 to form a ligase complex that suppresses the expression of photoprotective genes in the dark [23]. However, the molecular roles of CrCULs underlying the lipid accumulation process are still a puzzle. Here, *Cullin* genes from *C. reinhardtii* were identified and then their gene expression profiles under nutrition deprivation were revealed. The RNAi expression vectors for three *CrCUL*s were constructed and inserted into *C. reinhardtii* genomic DNA to obtain the gene inhibiting expression. Lipid contents and fatty acid (FA) profiles of *CrCUL* RNAi lines were surveyed to find out whether lipid biosynthesis in *C. reinhardtii* could be affected by CrCULs.

## 2. Results and Discussions

### 2.1. Cullins in C. Reinhardtii

The alignment sequences of the cullins E3 ubiquitin ligases (ID: PF00888) were obtained after searching in PFAM. Through Hmmsearch in *C. reinhardtii* proteome data with the cullins proteins as the queries, three CrCUL total sequences were obtained. Final cullins proteins obtained from the *C. reinhardtii* proteome in the Phytozome database were named as CrCUL2 (Cre17.g734400.t1.1), CrCUL3 (Cre07.g324050.t1.2) and CrCUL4 (Cre12.g516500.t1.1) based on the potential orthologs in Arabidopsis. The protein sequence characteristics are shown in Table 1. The length of the predicted CrCUL proteins were in the range of 736 (CrCUL3) and 766 aas (CrCUL4) and ORF lengths ranged from 2211 to 2301 bp. Moreover, the theoretical isoelectric point (pI) and the molecular weight of the CrCULs ranged from 6.98 to 8.49 and 84.8 to 87.6 kDa, respectively. Three CrCULs were hydrophilic proteins; among them, CrCUL4 and CrCUL2 had one and three possible transmembrane helices, respectively, while CrCUL3 had no transmembrane domain.

In recent years, cullin proteins in higher plants and animals have been well understood. Their special features and various functions have been well characterized. However, cullin proteins in green microalga were little known of to date. In this study, the *cullin* gene family in the *C. reinhardtii* genome was investigated by using cullin alignment sequences as queries. A total of three *CrCUL* homologue genes were isolated from the *C. reinhardtii* genome sequence and the gene numbers were different from those in higher plants but similar to those in fungi. An early phylogenetic analysis showed all CUL proteins were derived from three ancestral genes, *CULα*, *CULβ* and *CULγ*. Three identified *CrCUL*s in the *C. reinhardtii* genome confirmed the primitive evolution status of *C. reinhardtii*. *CrCUL* genes are located on different chromosomes, which are similar to the *CUL*s in *S. cerevisiae*. The lengths of CrCULs are 736 to 766 amino acids, which are similar to AtCULs. The length of the predicted ORFs and the amino acid sequences of all CrCULs are similar to CULs characterized in Arabidopsis [24]. Furthermore, both CrCUL2 and CrCUL4 have transmembrane helices, indicating that they might participate in molecular transmembrane transport.

**Figure 2:**
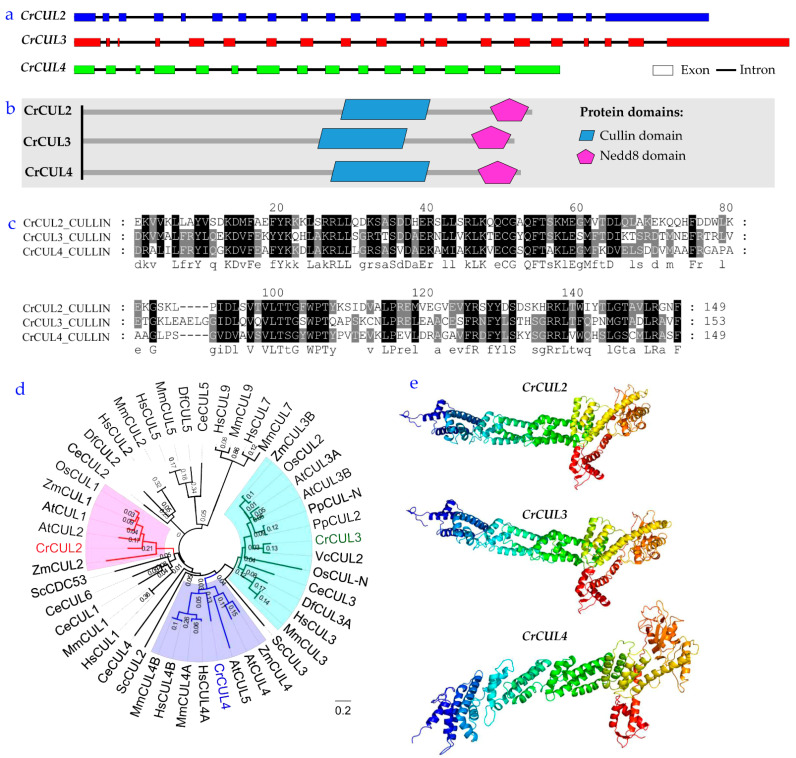
Sequence analysis of cullins in *C. reinhardtii*. (**a**) Gene structures of *CrCUL*s; colored boxes indicate exons and lines show introns. (**b**) Conserved motif composition of CrCULs. The blue and purple boxes indicate cullin and nedd8 domains, respectively. (**c**) Sequence alignment of cullin domains of CrCULs using MUSCLE. Numbers show the positions of amino acid residues. Conserved residues are shown in black. (**d**) A phylogenetic tree of CrCULs was constructed using MEGA7.0. The phylogenetic tree was created by a neighbor-joining method and used 1000 bootstraps on the basis of the amino acid sequence of cullins from model organisms. Cr, *Chlamydomonas reinhardtii*; Hs, *Homo sapiens*; Mm, *Mus musculus*; Df, *Drosophilid fly*; Ce, *Caenorhbditis elegans*; Vc, *Volvox carteri*; Sc, *Saccharomyces cerevisiae*; At, *Arabidopsis thaliana*; Os, *Oryza sativa*; Zm, *Zea mays*; Pp, *Physcomitrella patens*. (**e**) 3D model of CrCULs predicted by the Phyre2 software.

Exons/intron structures of *CrCUL* genes were visualized by DAMMAN. As shown in Figure 2a, the three *CrCUL*s contained 14–19 introns showing the complexity of the *C. reinhardtii* genome. All CrCUL proteins have domains typical for the cullin family, which include a cullin domain and an N-terminal Nedd8 domain (Figure 2b). A cullin domain at the C-terminal ends of cullins binding to RING proteins (Rbx1/ROC1) serves as the catalytic centers of CRL complexes while the Nedd8 domain at the N-terminal end promotes CRL activity through neddylation [25]. To examine the cullin domain features of CrCUL proteins, the sequence alignment was performed using MUSCLE (Figure 2c). Conserved amino acid residues of a cullin motif of CrCULs were highly homologous. These findings suggested that CrCULs encoded the cullins protein family in *C. reinhardtii*.

The phylogenetic tree based on the cullins protein sequence showed that cullins were evolutionarily conserved from human to yeast. CrCUL2 and CrCUL3 proteins clearly belong to the plant cullins branch (Figure 2d). However, CrCUL4 protein as homologous with the animal cullins. A phylogenetic tree analysis revealed that CrCUL2 and CrCUL3 might have similar functions with CULs in plants, which play key roles in the control of photomorphogenesis and multiple developmental pathways [26]. The 3D model of CrCULs was constructed by Phyre2 based on a template human Cullin4a (PBD ID: c2hyeC, chain C, resolution of 7.40 Å) [27]. As shown in Figure 2e, CrCUL2 and CrCUL3 exhibited similar structures with the model identification of 53% and 39%, respectively, while CrCUL4 had β-sheets at the N-terminal with a model identification of 37%. The strong similarity between the predicted three-dimensional structure of CrCULs (Figure 2e) and the cullin and Nedd8 domains confirmed that CrCULs belong to the cullin protein family.

### 2.2. Expression Patterns of CrCUL Genes under Different Nutrition Deficiency Conditions

Previous studies showed that nutrition starvation was the most effective method to induce lipid accumulation in microalgae. Many key genes involved in lipid biosynthesis were revealed under nutrition deficiency conditions [28]. Here, in order to evaluate the expression patterns of *CrCUL* genes in response to nutrition deficiency, a quantitative real-time RT-PCR analysis with cells subjected to eight days of nutrition deprivation conditions such as nitrogen deficiency (-N), sulfur deficiency (-S) or iron deficiency (-Fe) was performed. The relative mRNA expression levels of *CrCUL*s in stress treatment groups were calculated by normalizing with a control.

**Figure 3:**
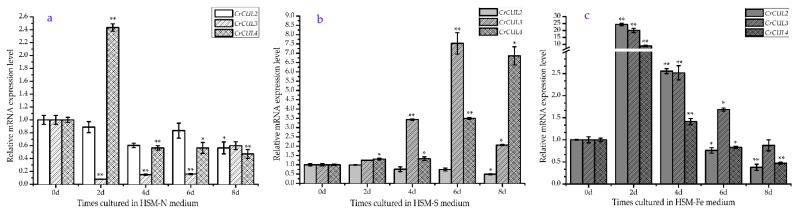
Expression patterns of *CrCUL*s in *C. reinhardtii* under deficiency stresses for eight days. The relative expression levels of *CrCUL*s under nutrition deprivation conditions including nitrogen (**a**), sulfur (**b**) and iron (**c**) deprivation were calculated. Each value represents the mean ± standard error (SE) of the three replicates. * and ** indicate significant differences *p* ≤ 0.05 and *p* ≤ 0.01, respectively, according to Dunnett’s analysis.

Nitrogen (N) and sulfur (S) are necessary macroelements for microalgal metabolism. The limitation of the supply of these elements has significant negative impacts on cell count, total protein and chlorophyll levels but lipid production increased by several folds more than the normal conditions [2]. Compared with the control, there was no change in the *CrCUL2* gene expression level after N-deficient treatment for six days but a decline was observed at eight days (Figure 3a). *CrCUL3* remarkably decreased and reached its lowest point (0.07-fold) after N was limited for two days then recovered gradually. The transcriptional level of *CrCUL4* was significantly induced (2.43-fold) by N deficiency after two days but inhibited thereafter. Nitrogen is one of the important elements for *C. reinhardtii*. Under N stress, photosynthetic pigments were progressively lost and photosynthetic apparatus parallel decreased [29]. CUL3 in Arabidopsis modulates the phototropic responsiveness that mediates mono/multiubiquitination of *phot1* [30]. CUL4 also participates in higher plant photo morphogenesis. The transcriptional level changes of *CrCUL3* and *CrCUL4* during N deficiency indicated that they might mediate *C. reinhardtii* photomorphism. On the other hand, in the case of S deficiency, the *CrCUL3* and *CrCUL4* genes were induced dramatically (Figure 3b). The transcriptional level of *CrCUL3* and *CrCUL4* had peaks after 6 d and 8 d, respectively, which were 7.5 times and 6.9 times higher than that of the control. By contrast, the *CrCUL2* gene was down-regulated by 49.2% after S deficiency treatment for eight days. Overall, *CrCUL2* seemed insensitive to N and S deficiencies and exhibited a decrease after treatment for eight days. *CrCUL3* obviously decreased under N deficiency but induced significantly under S deficiency (Figure 3a,b). Although CULs in higher plants have been isolated and their expression profiles under different lighting conditions have been identified in previous studies [31], a *CUL* gene expression analysis under nutrition deficiency is firstly revealed in this study.

It was interesting to note that *CrCUL* genes shared similar expression patterns under iron deficiency conditions, which peaked after two days. The degrees of up-regulation then declined slightly (Figure 3c). Iron (Fe) is one kind of microelement for unicellular photosynthetic algae *C. reinhardtii*. Fe deficiency could obviously deduce the photosynthetic rate and photosynthesis-related proteins but could also induce the accumulation of TAG, which results in the formation of lipid droplets [32]. Previous studies have shown several members of Coat Protein Complex I (COP I) increased under metal deficiency/limitation conditions [33]. COP I is a WD40 repeat (WDR) domain containing E3 ubiquitin and often adapts with CUL4 to inhibit normal rates of lipid degradation [34]. However, the knowledge about the involvement of ubiquitin ligase under nutrition deficiency was limited. Here, dramatic increases in the level of *CrCUL*s under iron deficiency were observed (Figure 3c), which implied the relation between CrCULs and metabolism under iron deficiency.

### 2.3. RNAi Expression of CrCULs in C. reinhardtii

**Figure 4:**
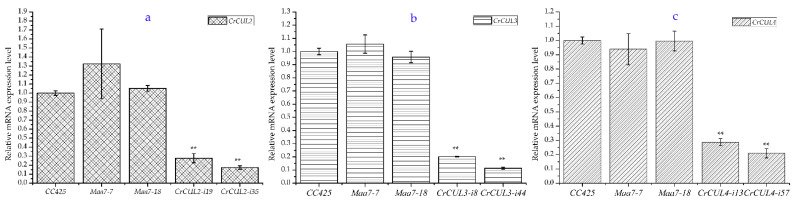
The expression levels of *CrCUL*s decrease in RNAi transformants. Relative mRNA expression levels of *CrCUL*s were analyzed by real-time RT-PCR analysis, (**a**) for the *CrCUL2* gene, (**b**) for the *CrCUL3* gene, (**c**) for the *CrCUL4* gene. Three experiments were performed with the *CC425* strain as the control. Error bars indicate the standard deviation. The asterisks show statistically significant differences by Dunnett’s multiple range test; ** *p* ≤ 0.01.

*C. reinhardtii CC425* was transformed with the p*Maa7IR* or p*Maa7IR*-*CrCUL* vectors in which dsRNA was generated from *CrCUL* cDNA fragments driven by a *CrRbcS* promoter. Positive transformants were grown under 5-fluoroindol (5-FI) and paromomycin selective pressures. The RNAi expression lines of *CrCUL*s were then determined by qRT-PCR randomly. The *CC425* strains and p*Maa7IR* transformants were applied as controls (named as *Maa7* lines). The mostly inhibited *CrCUL* lines were confirmed as the RNAi lines and chosen for a further analysis. According to the results of the qRT-PCR assays, *CrCUL* RNAi lines were successfully constructed (Figure 4). Compared with the *CC425* strains, the relative *CrCUL2* gene expressions in *CrCUL2*-i19 and *CrCUL2*-i35 lines decreased by 27.7% and 17.4%, respectively (Figure 4a). The related genes in the *CrCUL3*-i8 and *CrCUL3*-i44 strains were deprived by 20.2% and 11.3%, respectively (Figure 4b). The mRNA expressions of *CrCUL4* in the *CrCUL4*-i13 and *CrCUL4*-i57 lines were inhibited by 28.8% and 20.9%, respectively (Figure 4c) while related gene expressions in the p*Maa7IR* transformants (*Maa7*s) changed little. These data indicated that the *CrCUL* expression was significantly interfered with in the RNAi strains. RNAi strategies using a tandem inverted repeat (IR) system have been applied successfully in *Chlamydomonas* reverse genetic studies [35]. PEPC, DGAT2 and many other key enzymes in lipid synthesis have been characterized by the RNAi method [7]. The inhibitory efficiency of the above RNAi on mRNA levels of *CrCUL*s suggested that the transgenic lines were suitable for a further analysis.

### 2.4. Growth Kinetics and Lipid Contents of CrCUL RNAi Lines

**Figure 5:**
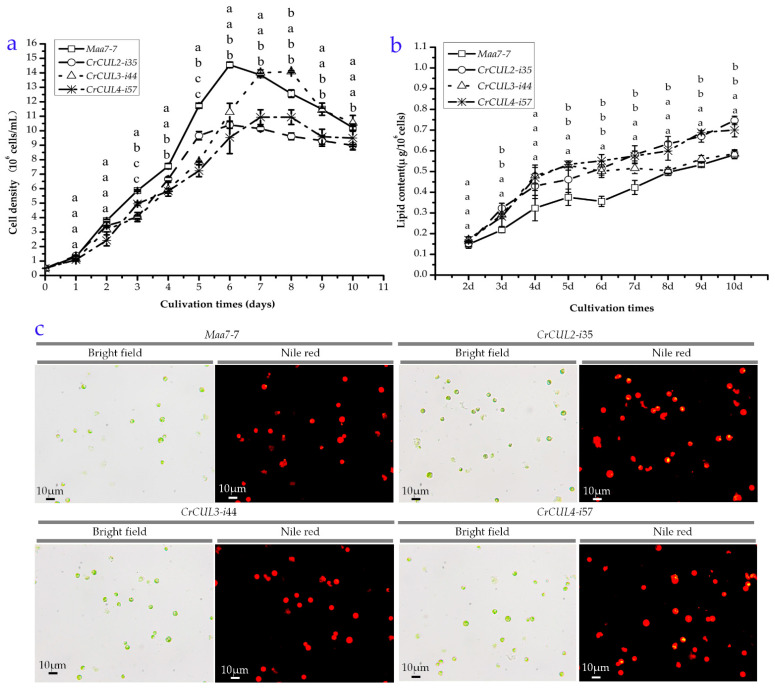
The growth and lipid accumulation of *CrCUL* RNAi lines. (**a**) The growth curve of *CrCUL* RNAi lines. Different letters indicate significant differences (*p* ≤ 0.05) by Dunnett’s multiple range test. (**b**) Total lipid contents of *CrCUL* RNAi lines. The lipid content of each strain was normalized to cell densities quantified at the growth phase. The data are representative of three replicas and different letters indicate significant differences (*p* ≤ 0.05) by a one-way ANOVA. (**c**) TAG disturbers in *CrCUL* RNAi lines and the control. Cells were grown in an HSM medium for 10 days. Lipid bodies were stained with Nile red (yellow dots) and imaged by fluorescence microscopy. Bar = 10 µm.

To evaluate the effect of *CrCUL* gene silencing on cell growth, the successful *CrCUL* RNAi lines were selected for a growth kinetics analysis with the empty vector transformant (*Maa7*-7 line) as the control. A similar seedling density (5 × 10^5^ cells/mL) and equivalent sampling intervals were applied at the beginning. The cell density was measured during growth phase per day. After being cultured for three days, *CrCUL* mutants showed decreased cell densities, as shown in Figure 5a. Mutants *CrCUL3*-i44 and *CrCUL4*-i57 showed growth peaks at day seven while *CrCUL2*-i35 and the control showed growth peaks at day six. After being grown for seven days, the *CrCUL2*-i35 and *CrCUL4*-i57 strains showed 28% and 34% less cell density than the control (Figure 5a), respectively, while the biomass productivity of *CrCUL3*-i44 increased 1.12-fold compared with the *Maa7*-7 strain at day eight. Although the *CrCUL3* and *CrCUL4* mutants showed varied growth profiles, their cell numbers became similar after 10 days of growth. Therefore, RNA silencing of *CrCUL* genes showed a negative effect on cell growth.

CRLs are major regulators of the cell cycle. For example, CRL1 complexes control cell proliferation and cell cycle progression by degrading cell cycle regulators binding to F-box proteins (FBP). Cul4-DDB1 ubiquitin ligase interacts with and degrades DNA replication factor Cdt1 controlling DNA replication [36]. CUL2 interacts with VHL (von Hippel-Lindau) and is required for a cell cycle G1-S period transition and for mitotic chromosome condensation [37]. At the same time, CUL4a CRL monoubiquitylates histone H2A acting in chromatin remodeling and CRL4 responds to DNA damage in metazoans and plants. Moreover, CRL3 binds to the BTB protein to participate in cell differentiation but not the cell cycle. The inhibition of CrCUL2 and CrCUL4 resulted in reduced cell numbers (Figure 5a) possibly because of the important roles of CUL2 and CUL4 in regulating the cell cycle. However, we were unable to speculate the reason for the irregular cell numbers of the *CrCUL3* RNAi lines.

CRLs have been demonstrated to control the formation of lipid droplets in mammals in past decades. In an attempt to understand the role of CrCULs in lipid synthesis regulation, we examined the lipid content in *CrCUL* RNAi strains. After two days of culture under similar environmental conditions, cells were collected for a lipid content analysis. Lipids in cells were stained by Nile red. The fluorescence intensity was converted into the lipid concentration according to the standard curve and then normalized by the cell density, as shown in Figure 5b. All three *CrCUL* gene silencing strains increased in lipid content compared with the control (*Maa7* empty vector transformant). However, the silencing mutant of *CrCUL3* exhibited a different curve pattern of a dynamic lipid content from *CrCUL2* and *CrCUL4*. Compared with the control, the silencing mutants of *CrCUL2* and *CrCUL4* constantly encouraged lipid biosynthesis in cells but the silencing mutant of *CrCUL3* did not exhibit any promotive effect on lipid content from day seven. The silencing mutants of *CrCUL2* and *CrCUL4* accumulated the highest lipid levels at day ten; approximately 1.28 and 1.20 times more than the control, respectively. By contrast, the first peak value of lipid content occurred at day five and a putative stationary phase of lipid content occurred thereafter in the silencing mutant of *CrCUL3*. Therefore, a conclusion that the biomass was inversely associated with the lipid contents in *CrCUL* mutants could be drawn. However, the role of CrCUL3 seems to be different from CrCUL2 and CrCUL4.

To observe the lipids in *CrCUL* RNAi lines, Nile red was used to bind to neutral lipids such as TAG in cells. The imaging results indicated that *CrCUL2*-i35 and *CrCUL4*-i57 cells accumulate more TAG than the control cells (Figure 5c), which agreed with the higher lipid contents detected by the fluorescence intensity. It was direct evidence for CULs participating in lipid formation that CUL3 in LiSa-2 cells localized around LDs and the down-regulation of CUL3 and the inhibition of neddylation could block the formation of lipid droplets (LDs) [38]. However, after silencing the gene expression of *CrCUL*s, the *CrCUL3* RNAi lines did not show constantly tremendous changes in lipid content. A possible reason is that CULs from plants and animals have different roles. Although the functions of CULs in higher plants have been demonstrated intensively, the information of CULs in microalgae is very limited. So far, the photoprotective role of the E3 ubiquitin ligase CUL4-DDB1-DET1 complex, which mediates blue-light signals to the LHCSR1 and LHCSR3 gene expression in *C. reinhardtii*, has been determined by using a specific inhibitor for CRL4 [23]. Stress-response proteins LHCSR1 and LHCSR3 also could be induced by nutrient starvation, which is beneficial for lipid accumulation. The lipid metabolic regulation function of CrCUL2 and CrCUL4 found in this study is an important supplement for CRLs in microalgae. However, it still requires further investigation on how CUL proteins work.

### 2.5. Fatty Acid Composition Analysis

Fatty acid (FA) composition is an important factor for biodiesel production. To illustrate the FA composition in the *CrCUL* RNAi lines, GC-MS was used to analyze the FA contents in transformants after 10 days of culture (Table 2). The highest total fatty acid (TFA) content was detected in the *CrCUL4*-i57 line, i.e., 75.30 μg/mg, followed by *CrCUL2*-i35 and *CrCUL3*-i44, which were 62.24 and 58.04 μg/mg, respectively. Only 55.19 μg/mg of TFA was detected in the control samples. Compared with the control group, TFA in *CrCUL2*-i35, *CrCUL3*-i44 and *CrCUL4*-i57 increased by 12.8%, 5.2% and 36.4%, respectively. The FA contents of *CrCUL3*-i44 did not change significantly (*p* > 0.05), similar to its lipid contents. The increase in TFA content of the *CrCUL2*-i35 strains was contributed by the increase of C16:0 and C18:0 fatty acids, which increased by 119.8% and 117.1%, respectively, compared with the control. C16:2 in the *CrCUL2*-i35 cells decreased by 5.0% compared with the control. In the most TFA strain *CrCUL4*-i57, C16:0, C18:0, C18:3 (5, 9, 12), C18:3n3 and C20:0 had increases ranging from 130.7% to 154.15%. The results indicated that silencing *CrCUL2* and *CrCUL4* not only altered the TFA contents but also changed the FA composition.

The modification of the FA synthesis pathways in microalgae was thought to be an effective strategy to improve the productivity of lipid synthesis. Research aimed at elevating the contents of fatty acids in microalgae strains for biofuel production have been performed for many years. Microalgae rate-limiting enzymes of an FA synthesis were analyzed and transferred to encourage lipid accumulation [9]. Nutritional polyunsaturated fatty acids (PUFAs), which are an important byproduct of microalgae, also need to be induced by genetic engineering. More recently, CRLs were reported to participate in fatty acid metabolism that a CUL3-MATH-BTB/POZ E3 ligases complex interacted with ETHYLENE RESPONSE FACTOR (ERF)/APETALA2 (AP2) transcription factors and thus altered fatty acid contents in Arabidopsis seeds [39]. Fatty acids C16:0 and C18:0, which are the main components for the preparation of biodiesel, were shown to be induced when *CrCUL2* and *CrCUL4* were silenced. Correspondingly, our results also demonstrated that *CrCUL2* and *CrCUL4* RNAi induced the accumulation of TFA and lipids (Figure 5 and Table 2). Based on the above results, we proposed that CrCUL2 and CrCUL4 might function as negative regulators in lipid biosynthesis by degrading a few key enzymes. In future, identifying the signaling pathways involving CrCRLs will be required.

### 2.6. Subcellular Localization of CrCUL4

**Figure 6:**
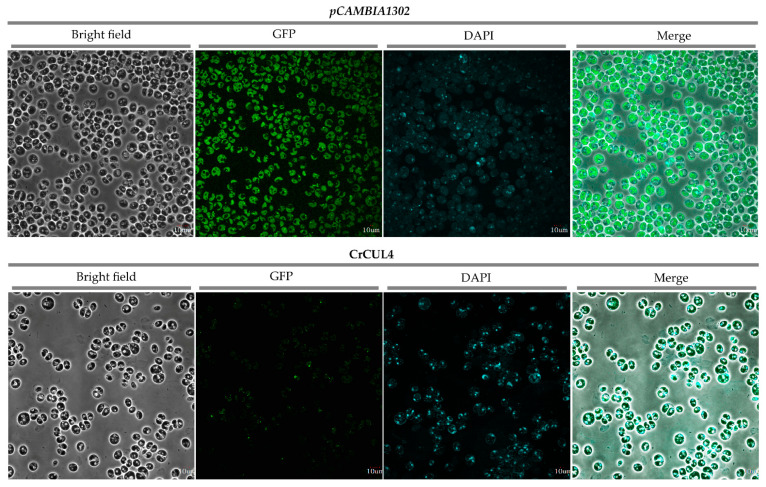
Localization of the CrCUL4-GFP protein. Individual panels show corresponding bright-field images, CrCUL4-GFP in *C. reinhardtii CC124* cells and DAPI stained images and merged images, respectively. CrCUL4-GFP fusion was driven by the *CaMV 35S* promoter in vector p*CAMBIA1302*. Bars = 10 μm.

CUL4-based E3 ligases (CRL4s) composed of CUL4, DDB1 and one WD40 protein have diversly important functions in cells. To determine its subcellular localization, CrCUL4 was inserted into a frame of a GFP reporter gene under the control of the cauliflower mosaic virus 35S (*CaMV 35S*) promoter. The recombinant *CrCUL4-GFP* fusion gene or the empty vector of p*CAMBIA1302* were introduced into the *C. reinhardtii* CC124 strain by agrobacterium infection using kanamycin as the screening pressure. The positive colonies were chosen and observed by Olympus laser scanning confocal microscopy (LSCM). As shown in Figure 6, the CrCUL4-GFP fusion protein accumulated mainly in the nucleus whereas GFP alone was present throughout the whole cell, suggesting that CrCUL4 was a nucleus localized protein. In animals, CUL4 formed as a CRL4 complex, which is crucial for DNA replication and the cell cycle [40]. CUL4 conjugating with DDB1 in higher plants is located in the nuclear and is involved in multiple plant developmental processes [41]. A similar result in this study (Figure 6) showed that CrCUL4 may act at a chromatin level.

## 3. Materials and Methods

### 3.1. In Silico Identification and Sequence Analysis of CrCULs

The seed sequences of alignments of the cullin protein family were identified in the PFAM (https://pfam.xfam.org/, accessed on 28 April 2021) database by searching with cullin as a keyword. An Hmmsearch was performed in HMMER (https://www.ebi.ac.uk/Tools/hmmer/search/hmmsearch, accessed on 28 April 2021) *C. reinhardtii* proteomes used cullin proteins as query sequences, setting the E-value to less than 1 × 10^−50^. Finally, all *C. reinhardtii* cullin sequences obtained from Hmmsearch were analyzed by BLASTp using version 5.5 of the *C. reinhardtii* proteome obtained from the Phytozome V12.1 database (https://phytozome.jgi.doe.gov/pz/portal.html, accessed on 28 April 2021). Only the sequences with an E-value cutoff of 1 × 10^−50^ were chosen as the candidate genes.

The intron/exon boundaries of *CrCUL* genes were shown by DNAMAN software. The motif analysis of the CrCUL proteins were verified by the SMART program (http://smart.embl-heidelberg.de/, accessed on 28 April 2021). Sequences of homologous proteins from model organisms were retrieved from NCBI (https://www.ncbi.nlm.nih.gov/, accessed on 28 April 2021) by blastp searching. A NJ phylogenetic tree of CrCULs was generated by Mega7.0 using a MUSCLE alignment with a bootstrap of 1000 replicates [42]. The phylogenetic tree was edited by FigTree v1.4.3 (http://tree.bio.ed.ac.uk/software/figtree/, accessed on 28 April 2021). The protein properties of the CrCULs including molecular weight, isoelectric point and hydrophobic properties were analyzed by ExPASy (https://www.expasy.org/, accessed on 28 April 2021). The transmembrane regions and orientation were predicted by TMpred (https://embnet.vital-it.ch/software/TMPRED_form.html, accessed on 28 April 2021). Comparative 3D protein predictions were performed using the Phyre2 (http://www.sbg.bio.ic.ac.uk/phyre2/html, accessed on 28 April 2021).

### 3.2. C. reinhardtii Strain and Growth Conditions

*C. reinhardtii CC425* (cell wall deficient strain) was obtained from the College of Life Sciences and Oceanography of Shenzhen University (Shenzhen, China). Cells were grown under the following conditions: continuous illumination with an intensity of 25 μmol photons·m^−2^·s^−1^ in 250 mL conical flasks containing 1/3 Tris-acetate-phosphate (TAP) media at 24 °C. For the gene expression analysis of *CrCUL*s, cells in the logarithmic phase were collected and washed twice with fresh water by centrifugation at 3000× *g* for 5 min. After being washed, cells were transferred into either a low-nitrogen (-N), low-sulfur (-S) or low-iron (-Fe) medium grown for eight days and Sueoka’s high salt medium (HSM) culture served as the control. The components of all media are listed in Table S1. Cells were collected by centrifugation, immediately frozen in liquid nitrogen after harvesting and then stored at −80 °C until use.

For the transgenic experiment, cells were cultured to the logarithmic phase then collected and resuspended with a fresh TAP medium. Gene transfection was performed by the glassbeads method as described by Kindle et al. [43] and, after dark repair for 48 h, cells were plated on the selection media.

### 3.3. Gene Expression Analysis of CrCULs

Total RNA was isolated from -N, -S or -Fe cultured cells using a Trizol reagent (Invitrogen, Carlsbad, CA, USA). Genomic DNA contamination was erased and a first-strand cDNA synthesis by a PrimeScript™ RT reagent kit with a gDNA Eraser (Takara, Kyoto, Japan) was used. Real-time RT-PCR was carried out in an Agilent StrataGene M × 3005 (Agilent, Santa Clara, CA, USA) using a SYBR Premix Ex Taq Kit (Takara, Kyoto, Japan). The internal reference gene was set as the housekeeping gene *18S rRNA* (Genbank No: MF101220.1). The relative gene expression levels were calculated using the 2^−ΔΔCt^ method. Three biological replicates were performed for all relative abundances of an mRNA level analysis. All primers are listed in Table S2.

### 3.4. RNA Interference (RNAi) Vector Construction and Algae Transformation

To clone *CrCUL* genes, primer pairs (Table S2) were designed by Primer Premier 5.0 according to the predicted *CrCUL* coding sequences (CDS) obtained from the *C. reinhardtii* genome, version 5.5, in the Phytozome database. The cDNAs were generated from the total RNA of four day cultured cells with oligo dT primers using a PrimeScript™ RT-PCR kit (Takara, Kyoto, Japan). A PCR was performed using the cDNA as the template. PrimeSTAR Max DNA Polymerase was applied with a protocol of 94 °C for 5 min, 35 cycles of 94 °C for 30 s, 56 °C for 1 min, 72 °C for 3 min 30 s and 72 °C for 10 min. The PCR products were purified and cloned into a pMD18-T vector (Takara, Kyoto, Japan) and then sequenced by universal primers. The CDS of the *CrCUL4* gene were constructed into the p*CAMBIA*1302 vector and transformed into the *CC124* for a subcellular location analysis. The positive colonies were observed by Olympus laser scanning confocal microscopy (Olympus, Kyoto, Japan).

The RNAi mediated silencing vectors of the *CrCUL* genes were constructed as described previously [44]. In brief, a 388 bp cDNA of *CrCUL2*, a 324 bp cDNA of *CrCUL3* and a 251 bp cDNA of *CrCUL4* were amplified from *CrCUL* CDS cloned by using primer pairs listed in Table S2. These fragments were inserted into the p*Maa7IR* silencing vector in a sense orientation and anti-sense orientation with restriction digestion and ligation. *CrCUL* RNAi vectors were transformed into the *C. reinhardtii CC425* strain via the glass bead method. A TAP medium with 1.5 mM L-tryptophan, 5 μg/mL paromomycin and 5 μM 5-FI (Sigma-Aldrich, Saint Louis, MO, USA) was applied to select positive transgenic lines. The resistant colonies were picked up and the silencing efficiency of the *CrCUL* genes was detected by qRT-PCR.

### 3.5. Growth and Neutral Lipid Analysis

*CrCUL* RNAi transgenic strains and negative controls (p*Maa7IR*/*X* transgenic strains, shown as *Maa7* lines) were grown under normal conditions (HSM medium) as described above for 10 days. The cell density was measured using a Coulter™ Multisizer 4 (Beckman Coulter, Fullerton, CA, USA) every day.

For neutral lipid content (NL) examination, cells were stained by 0.5 µg/mL of Nile red (Sigma-Aldrich, Saint Louis, MO, USA) dissolved in 25% (*v*/*v*) of DMSO solution. The fluorescence intensity (FI) was measured with a Glomax-Multi Detection System (Promega, Madison, WI, USA) with excitation and emission wavelengths of 530 nm and 575 nm, respectively. Triolein (Sigma-Aldrich, Saint Louis, MO, USA) was used to make a standard curve. The neutral lipid content (NL) was calculated according to the formula:

NL (μg/10^6^ cells) = [(0.0004 × FI (530/575) − 0.0038] × 50/cell density (1)

The lipid accumulation in transgenic strains were observed by a Nikon 80 i fluorescence microscope (Nikon, Kyoto, Japan) after being stained with Nile red. The excitation and emission wavelengths of 480 nm and 580 nm were applied, respectively.

### 3.6. Fatty Acid Methyl Ester Profiling

After being cultured in HSM for 10 days, *CrCUL* RNAi lines and the controls were harvested by centrifugation and dried in a vacuum freezing machine. About 5–10 mg dry biomass were weighted for lipid extraction. First, 1 mL of a 2 M sodium hydroxide-methanol solution was added to cells, vigorously mixed by a shaker for 1 h and then saponified at 75 °C for 15 min. A total of 1 µg of methyl nonadecanoate (C15:0) was added as an internal standard for a gas chromatography-mass spectrometry (GC-MS) analysis. For fatty acid methyl-esterification, 1 mL of 4 M hydrochloric acid–methanol was added and incubated at 75 °C for 15 min. After cooling down, 1 mL of hexane was added into the vial to extract the methyl esters. The organic eluent was concentrated with nitrogen, diluted in 500 μL of CH_2_Cl_2_ and then analyzed by GC/MASS Agilent 6890N (Agilent, Santa Clara, CA, USA).

### 3.7. Statistical Analysis

For comparisons of the means of *CrCUL* RNAi strains with the controls, a one-way analysis of variance (ANOVA), followed by a Dunnett’s test was performed using the SSPS11.5 program. Data are shown as mean ± SD from three independent experiments unless specified otherwise. Significant differences between the controls and other samples are indicated as follows: the * indicate that they were significantly different at *p* ≤ 0.05 and ** indicated *p* ≤ 0.01 according to one-way ANOVA.

## 4. Conclusions

In summary, we demonstrated for the first time the regulating roles of three CrCULs in lipid synthesis in *Chlamydomonas*. A phylogenetic analysis showed CrCUL2 and CrCUL3 were homologous with plant CULs but CrCUL4 was more like the animal CULs. All *CrCUL*s were transcriptionally induced by Fe deficiency for two days. The gene expression of *CrCUL*2 seemed to be insensitive to N and S deficiencies for six days. CrCUL3 was down-regulated by N-deficient conditions but up-regulated by S deficiencies. *CrCUL*4 expression was induced at two days after N-deficient treatment and then inhibited thereafter. After efficiently suppressing the expression of *CrCUL* genes by RNA interference, the RNAi of the *CrCUL2* and *CrCUL4* genes displayed a lower biomass but higher levels of lipid and TFA contents than the control. The significant alteration of lipid content and the composition of *CrCUL* mutants indicated that CrCULs play an important regulation role in lipid accumulation.

## Figures and Tables

**Table 1:** List of three *CrCUL* genes identified in *C. reinhardtii* and their sequence characteristics.

Gene Name	Chromosome Position	Introns	ORF	Deduced Polypeptide				
				Amino Acids	MW (kDa)	pI	GRAVY	Transmembrane Helices
*CrCUL2*	17:4895502…4903556F	19	2244	747	87.6	6.98	−0.214	3
*CrCUL3*	7:1516277…1525175F	18	2211	736	84.8	8.14	−0.473	0
*CrCUL4*	12:3957943…3963740F	14	2301	766	84.8	8.49	−0.576	1

ORF, open reading frame; MW, molecular weight; GRAVY, grand average of hydropathicity.

**Table 2:** FA content (μg/mg) of *C. reinhardtii CrCUL* RNAi lines.

FA	*Maa7*-7	*CrCUL2*-i35	*CrCUL3*-i44	*CrCUL4*-i57
C12:0	0.04 ± 0.01	0.04 ± 0.00	0.03 ± 0.00	0.04 ± 0.00
C14:0	0.04 ± 0.01	0.12 ± 0.11	0.04 ± 0.00	0.05 ± 0.01
C16:0	12.25 ± 0.65 ^a^	14.68 ± 0.89 ^b^	11.28 ± 1.60 ^a^	16.77 ± 1.47 ^c^
C16:1	3.95 ± 0.11	3.45 ± 0.63	2.01 ± 0.68	4.55 ± 2.21
C16:2	1.21 ± 0.12 ^a^	1.15 ± 0.20 ^b^	2.00 ± 0.03 ^a^	1.35 ± 0.19 ^a^
C16:3	1.08 ± 0.36	0.80 ± 0.06	1.29 ± 0.11	1.35 ± 0.27
C16:4	4.68 ± 0.19	6.35 ± 2.53	7.66 ± 0.85	7.37 ± 2.04
C18:0	1.40 ± 0.12 ^a^	1.64 ± 0.09 ^b^	1.27 ± 0.09 ^a^	1.83 ± 0.15 ^b^
C18:1n9t	4.10 ± 0.40	4.77 ± 0.51	3.12 ± 1.13	4.44 ± 2.90
C18:1n9c	5.50 ± 0.54	5.88 ± 0.89	4.39 ± 0.56	6.85 ± 2.37
C18:2	5.74 ± 0.23	6.27 ± 0.52	7.45 ± 0.98	7.31 ± 1.59
C18:3 (5,9,12)	6.30 ± 0.32 ^a^	6.47 ± 0.25 ^a^	5.56 ± 0.74 ^a^	9.65 ± 0.51 ^b^
C18:3n3	8.20 ± 0.63 ^a^	9.67 ± 2.35 ^a^	10.81 ± 1.36 ^a^	12.64 ± 3.16 ^b^
C20:0	0.42 ± 0.03 ^a^	0.50 ± 0.10 ^a^	0.55 ± 0.03 ^a^	0.63 ± 0.15 ^b^
C20:1	0.26 ± 0.03 ^a^	0.47 ± 0.31 ^a^	0.60 ± 0.03 ^b^	0.44 ± 0.16 ^a^
Total FA	55.19 ± 0.92 ^a^	62.24 ± 2.78 ^b^	58.04 ± 2.11 ^a^	75.30 ± 2.00 ^c^

^a,b,c^: Different letters of each line in the superscript position indicates they were significantly different at *p* ≤ 0.05 according to a one-way ANOVA.

## Data Availability

Not applicable.

## Supplementary Materials

The following tables are available online at https://www.mdpi.com/article/10.3390/ijms22094695/s1.

## Abbreviations

CRLs	Cullin-RING E3 ligases
CUL	Cullin
*C. reinhardtii*	*Chlamydomonas reinhardtii*
RNAi	RNA interference
TAG	Triacylglycerol
PDAT	Diacylglycerol acyltransferase
DGAT	Diacylglycerol acyltransferase
GPAT	Glycerol-3-phosphate acyltransferase
GPDH	Glycerol-3-Phosphate Dehydrogenases
Fat	Acyl-ACP thioesterase
ACCase	Acetyl-CoA carboxylase
PEPC	Phosphoenolpyruvate carboxylase
UPP	Ubiquitin proteasome pathway
E3s	E3 ubiquitin ligases
LD	Lipid droplets
SRS	Substrate-recognition subunit
SKP1	S-phase kinase-associated protein 1
SOCS/BC	Suppressor of cytokine signaling/elongin-BC
BTB	Broad complex, Tramtrack, Bric-a-brac
DDB1	DNA damage-binding protein-1
RBX1	RING Box 1
PARC	PARkin-like cytoplasmic protein
FA	Fatty acid
TAP	Tris Acetate Phosphate medium
HSM	Sueoka’s High Salt Medium
-N	Nitrogen deficiency
-S	Sulfur deficiency
-Fe	Iron deficiency
COP I	Coat Protein Complex I
WDR	WD40 repeat domain
IR	Tandem inverted repeat
GFP	Green fluorescent protein
