# Supplementary material for: The Roles of Cullins E3 Ubiquitin Ligases in the Lipid Biosynthesis of the Green Microalgae Chlamydomonas reinhardtii
Source: Int J Mol Sci. 2021 Apr 29;22(9):4695. doi: 10.3390/ijms22094695 (PMC8125325; doi:10.3390/ijms22094695)
Supplement: Supplementary file 1 [file ijms-22-04695-s001.zip › ijms-1194646-supplementary.pdf]

Supplementary Materials:

The Roles of Cullins E3 Ubiquitin Ligases in the Lipid Biosynthesis of the Green Microalgae *Chlamydomonas Reinhardtii*

Qiulan Luo [1], Xianghui Zou [1], Chaogang Wang [2], Yajun Li [3] and Zhangli Hu [2,*]

Table S1. Composition of the different media used (mg/L).

Components	TAP	HSM	HSM-N	HSM-S	HSM-Fe
Sodium acetate (hydrate)	-	2000	2000	2000	2000
Acetate	1049.00	-	-	-	-
Tris-Base	2420.00	-	-	-	-
K_2HPO_4	119.00	1440	1440	1440	1440
KH_2PO_4	61.00	720	720	720	720
NH_4Cl	400.00	500	546.7 (NaCl)	500	500
$MgSO_4{\cdot}7H_2O$	100.00	20	20	16.5 ($MgCl_2{\cdot}6H_2O$)	20
$CaCl_2{\cdot}2H_2O$	50.00	10	10	10	10
$FeSO_4{\cdot}7H_2O$	5.00	5.00	5.00	5.00	2.46 ($FeCl_2{\cdot}4H_2O$)
$Na_2{\cdot}EDTA$	50.00	50.00	50.00	50.00	50.00
$CoCl_2{\cdot}6H_2O$	1.61	1.61	1.61	1.61	1.61
$MnCl_2{\cdot}4H_2O$	5.00	5.00	5.00	5.00	5.00
H_3BO_3	11.40	11.40	11.40	11.40	11.40
$ZnSO_4{\cdot}7H_2O$	22.00	22.00	22.00	10.60 ($ZnCl_2$)	22.00
$CuSO_4{\cdot}5H_2O$	1.60	1.60	1.60	0.87($CuCl_2$)	1.60
$Na_2MoO_4{\cdot}2H_2O$	2.00	2.00	2.00	2.00	2.00

TAP, Tris Acetate Phosphate medium; HSM, Sueoka's High Salt Medium.

Table S2. Primers used in this work.

Purpose	Gene Name	Forward Primer(5′–3′)	Reverse Primer (5′–3′)
qPCR	*CrCUL*2	ACCCGGTAGCCGACATCTTC	CGCCACCGACTTGTTTACGA
	*CrCUL*3	GACCCGCAGTATGCAGACAA	GGCGTTACGGTACAGCTCCT
	*CrCUL*4	GTGACCTGGGCCGCCTGTAT	AGCCGCTCCACCATCTCCTT
Cloning	*CrCUL*2	ATGGCGGACCGCAAGCCTATCG	CTAGGCCAGGTACTTGAACAGG
	*CrCUL*3	ATGAAGAAGGGGCCGATTAAAA	TTAGGCCACGTAGGTATAGAAC
	*CrCUL*4	ATGTCAGCAGTAAGAGGCAAGG	TCAGGCGAGATAGTTGTACACG
RNAi-mediated vectors construction	*CrCUL*2	AACAAGTCGGTGGCGGGTAG	CGGACAGGTCAATGGGAAGC
	*CrCUL*3	GCGTTCGAGCACTTTGTGAA	GCTGGTCTTGATGTCCGTGA
	*CrCUL*4	CCGCATCAAGATCAACTCCA	CGTCTCGTGCCAGATACTCG
